# A Robust Molecular Rectifier Based on Ferrocene-Functionalized Bis(diarylcarbene) on Gold

**DOI:** 10.1021/acsami.4c20999

**Published:** 2025-02-17

**Authors:** Dandan Wang, Wenrui Xu, Yidan Hu, Tao Wang, Mark G. Moloney, Wei Du

**Affiliations:** a Oxford Suzhou Centre for Advanced Research, Building A, 388 Ruo Shui Road, Suzhou Industrial Park, Suzhou, Jiangsu 215123, P.R. China; b Institute of Functional Nano & Soft Materials (FUNSOM), Jiangsu Key Laboratory for Carbon-Based Functional Materials & Devices, Soochow University, 199 Ren’ai Road, Suzhou, Jiangsu 215123, P. R. China; c Chemistry Research Laboratory, Department of Chemistry, University of Oxford, Oxford OX1 3TA, U.K.

**Keywords:** molecular rectifier, ferrocene, molecular tunnel junction, carbene, surface modification

## Abstract

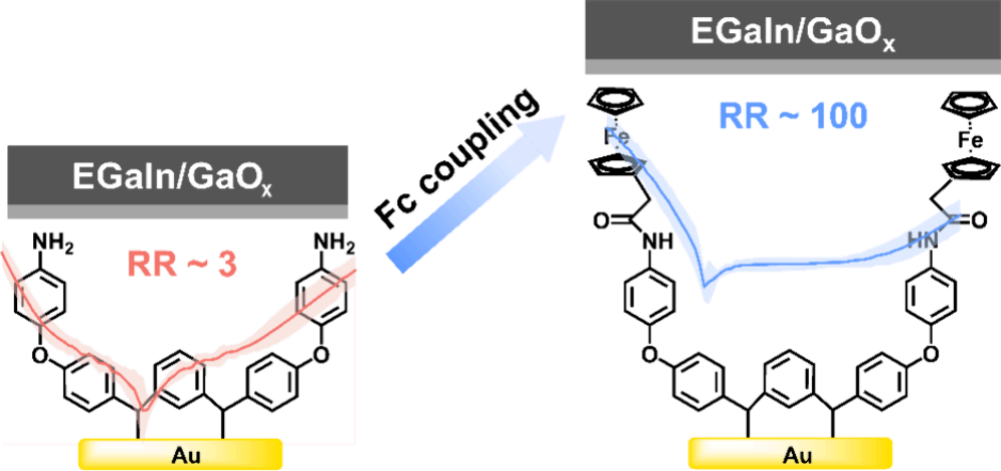

While thiol-based adsorbates have achieved significant success in surface modification and molecular electronics, their thermal and storage instability has hindered long-term commercial viability. Carbene-based thin films offer a promising alternative due to their enhanced stability; however, their molecular electronic properties after postfunctionalization remain to be investigated. In this study, by attaching a ferrocene (Fc) unit to a bis(diarylcarbene)-modified gold surface via carbodiimide coupling, the system exhibits diode behavior with a current rectification ratio of ∼100. This diode behavior is highly temperature-dependent, indicating that hopping is the dominant mechanism for current rectification. Notably, the system demonstrated excellent electrical stability under ambient storage conditions for over 6 months. Our work highlights the potential for designing carbene-based molecular junctions through postmodification and for developing durable functional molecular electronic devices.

## 1 Introduction

One of the appealing goals in molecular electronics is to design and fabricate molecule-based components as electronic devices whose properties and performance are complementary to semiconductor-based electronics.^1−5^ Over the years, an impressive variety of molecular devices such as rectifiers, switches, wires, transistors, or memristors have been developed through molecular structural control or molecule-electrode interface engineering.^6−14^ Among these molecular devices, a molecular rectifier, initially proposed by Aviram and Ratner in 1974,^15^ is comparatively simple and is one of the most fundamental components as a building block for diodes and transistors in molecular electronic circuits. Different types of molecular rectifiers have been explored, including those based on donor-spacer-acceptor compounds and molecules terminated solely with only donors or acceptors.^16−23^ Following the design principle of asymmetry introduction inside the metal–molecule–metal junction, molecules with redox active units are especially interesting and have been reported to provide a relatively high rectification ratio (RR = |*J*(*V*_fwd_)/*J*(*V*_rev_)|, where *V*_fwd_ and *V*_rev_ are the forward and reverse bias, respectively).^7,24−28^

Among various redox active groups, ferrocene (Fc) is one of the most widely studied functionalities owing to its straightforward synthetic chemistry for integration into diverse molecular architectures, as well as its well-studied electrochemistry.^2,29−32^ Generally, there are two different routes to covalently incorporate Fc into the molecular framework on a metal surface: by traditional direct synthesis and by chemical post modification. For the direct synthesis of Fc derivatives, self-assembled monolayers (SAMs) formed from thiols terminated with Fc have been extensively explored in large-area molecular junctions, giving a rectification ratio, which varies from ∼1 to ∼2 × 10^3^ at ±1 V on an Au surface,^7,27,33−35^ reaching ∼10^3^ at ±3 V on an Ag surface^16^ and ∼10^5^ at ±3 V on a Pt surface,^7^ with the exact value depending on factors such as molecular structure, monolayer quality, molecule–electrode interaction, substrate roughness, and thiol instability.^7,25,30,36−39^ For postmodification, azide-terminated alkylthiolated SAMs were formed and coupled with Fc through a copper-catalyzed azide-alkyne reaction; in this case, the rectification ratio varies from ∼82 to 166 at ±1.5 V.^28^ However, the sensitivity to oxygen (degradation in 1–2 weeks at room temperature), light, temperature (unstable >80 °C), and organic solvents (e.g., tetrahydrofuran) negatively impacts on the long-term stability of such thiol-based SAMs and significantly impedes commercial applications.^40−42^ To improve the stability of the monolayer, attempts to improve the molecular anchor to the metal surface have been made using bidentate or polymeric sulfur-based compounds^43,44^ and diselenide^45^ or carbene-derived molecules.^46^ Carbene ligands and especially N-heterocyclic carbenes (NHCs) have been increasingly employed to bind to metal surfaces over the last two decades.^46−54^ The Au-NHC bond is estimated to be ∼90 kJ/mol, stronger than an Au–S bond, and displaying superior chemical and thermal stability.^46,50,55,56^ However, previous experimental and theoretical studies have mostly been focused on the configurations (e.g., packing, orientation, and binding mode) of different NHCs on the metal surface, while functional devices such as carbene-based molecular rectifiers have rarely been reported.^57−59^

In this work, we fabricated carbene thin films with a bis(diarylcarbene) precursor, and Fc was then covalently attached to the carbene thin films via a one-step carbodiimide coupling. Once an Fc unit was coupled onto the carbene surface, charge transport across the Au-carbene-Fc interface was measured using an EGaIn (eutectic Ga/In) setup, showing a robust current rectification around 100 at ±2 V with high stability over 6 months. Our method could potentially be applied to integrate other functional groups into carbene-based molecular junctions, enabling stable molecular electronic devices with more functionalities.

## 2 Results and Discussion

### 2.1 Functionalization and Surface Characterization of the Au-Carbene-Fc Surface

**Figure 1:**
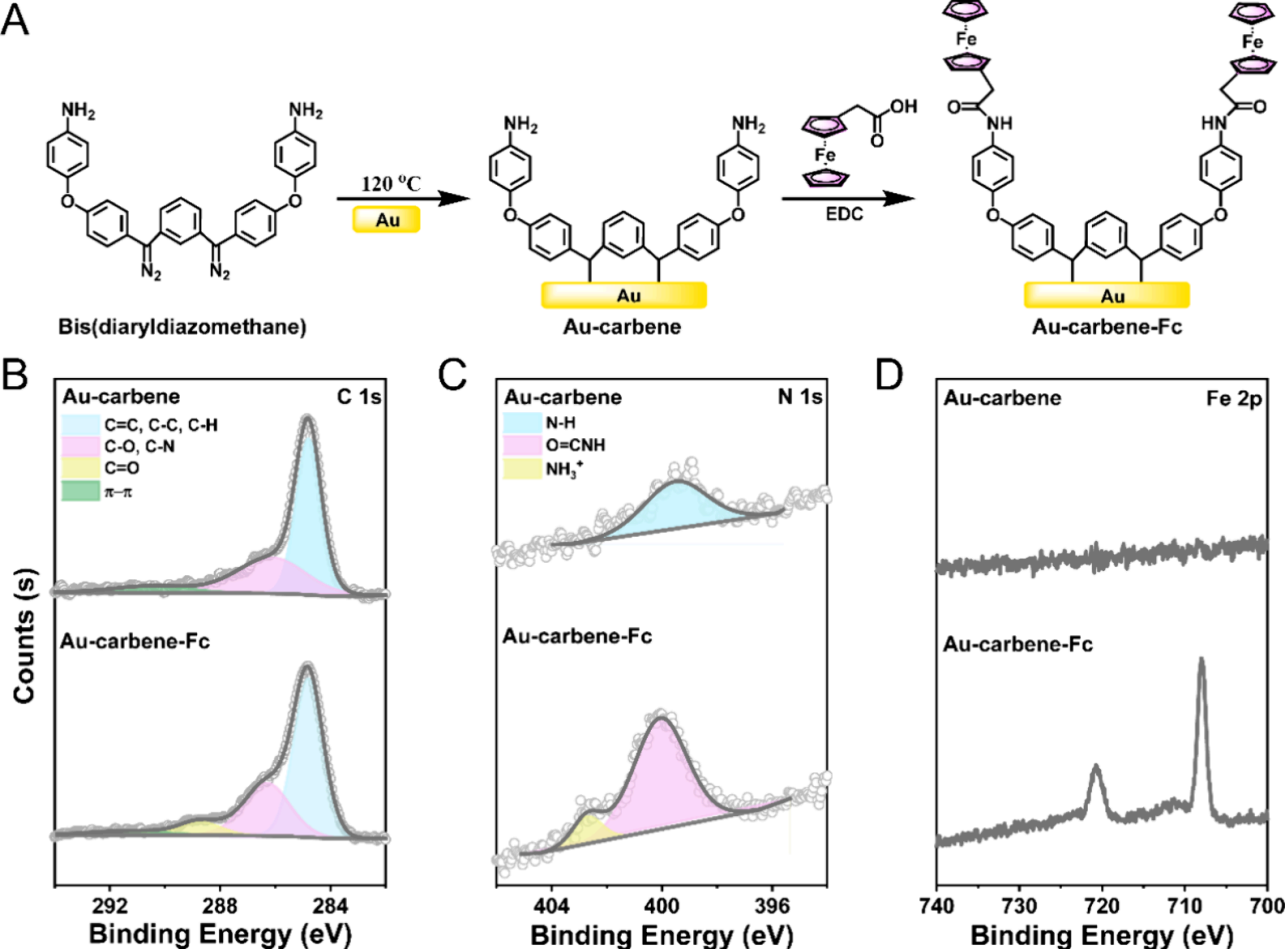
(A) Schematic illustration of the structure of Au-carbene and Au-carbene-Fc surface. (B–D) XPS high-resolution characterization of C 1s, N 1s, and Fe 2p for the Au-carbene (top curve) and Au-carbene-Fc surface (bottom curve). The bis(diaryldiazomethane) concentration *C*_diazo_ of 0.1 mg/mL was used as an example.

The bis(diarylcarbene) has been shown to form a C–Au bond with the gold surface,^49^ which is much stronger than the S–Au bond of thiolated SAMs. Here, we modified the Au surface at two different concentrations (denoted as *C*_diazo_) of bis(diaryldiazomethane), the precursor of bis(diarylcarbene), to generate an Au-carbene surface, followed by grafting ferroceneacetic acid onto the amine-terminated Au-carbene surface via carbodiimide coupling, to generate an Au-carbene-Fc surface (see Figure 1A for the surface structure). Although the amide unit was selected for its synthetic feasibility, it also has the ability to improve packing quality through lateral hydrogen bonding with similar tunneling behavior to alkyl units in thiolated molecular junctions.^60,61^ The bridging methylene group between Fc and the carboxyl group was important for reducing the steric hindrance during the coupling reaction; without it, the diimide coupling was found to be very inefficient. The elemental composition and chemical states of the surface before and after Fc attachment were evaluated by X-ray photoelectron spectroscopy (XPS). The XPS spectra for *C*_diazo_ = 0.1 mg/mL are shown in Figure 1B–D (the spectra for *C*_diazo_ = 0.2 mg/mL are provided in Figure S4). In the decomposed C 1s spectrum of Au-carbene, peaks at binding energies of 284.8, 286.3, and 290.3 eV are observed, attributed to C=C/C–C/C–H bonds, C–O/C–N bonds, and π–π shakeup contributions from aromatic rings, respectively. An additional peak at 288.6 eV in the Au-carbene-Fc spectrum (Figure 1B) corresponds to the C=O bond, suggesting the possibility of amide linkage formation after Fc coupling. Moreover, compared to the N 1s peak of the Au-carbene surface, which appears at 399.5 eV and is assigned to −NH_2_,^62^ a shift in the N 1s peak is observed for the Au-carbene-Fc surface(Figure 1C), which exhibits two peaks after decomposition. The dominant peak at 400.0 eV indicates −CONH– formation, suggesting the successful coupling of Fc. The shoulder peak at 402 eV suggests the presence of NH_3_^+^, possibly due to excess acidity during the coupling reaction. A control XPS experiment performed on a carbene layer treated with EDC alone revealed no significant EDC contamination on the carbene layer (Figure S5). Furthermore, the spectrum clearly reveals that the presence of the element Fe on the Au-carbene-Fc surface (Figure 1D), the Fe 2p_3/2_ peak dominating at 707 eV, along with a small shoulder at 711 eV attributed to the higher oxidation states (i.e., Fe^3+^), is consistent with the previous literature reports on ferrocenyl-alkanethiolated (SC_*n*_Fc) SAMs and postgrafted Fc thin films.^30,36,63^

### 2.2 Electrochemical Properties

**Figure 2:**
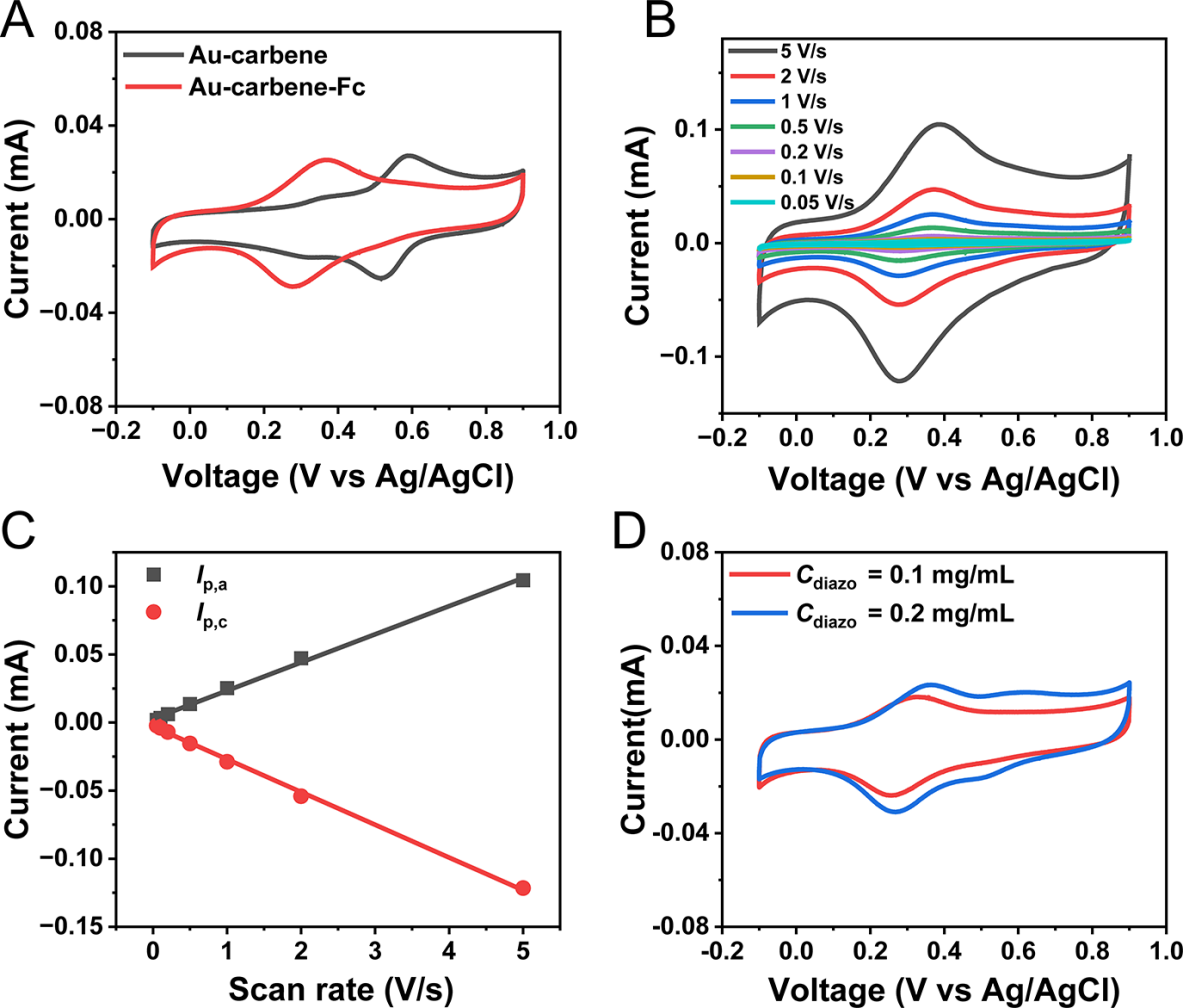
(A) Cyclic voltammograms recorded on Au-carbene (control) and Au-carbene-Fc surface with 1.0 M HClO_4_ as an electrolyte at a scan rate of 1 V/s. (B) CV recorded at different scan rate from 5 to 0.05 V/s. (C) The linear fitting of anodic and cathodic peak currents with the scan rate *v*, characteristic for electrode adsorbed species. Unless otherwise stated, *C*_diazo_ = 0.1 mg/mL was used throughout the experiments. (D) CV recorded on the Au-carbene-Fc surface with *C*_diazo_ increases from 0.1 to 0.2 mg/mL.

We compared the electrochemical behavior of Au-carbene and Au-carbene-Fc surface (Figure 2A, *C*_diazo_ = 0.1 mg/mL). For the Au-carbene surface, a distinct peak at 0.59 V and a small shoulder peak at 0.38 V for the oxidation potential are observed, likely originating from the oxidation of the aniline terminal group on the Au-carbene surface. This oxidation process is in agreement with reports for aminothiophenol SAMs on a gold surface,^64−66^ where the higher oxidation peak corresponds to the aniline dimer and the lower oxidation peak corresponds to the derived iminoquinone.^65^ Interestingly, after Fc attachment, the peak at 0.59 V almost disappears due to the coupling reaction, and a new broad peak at 0.33 V appears, which is the characteristic of the oxidation potential for ferrocene.^25,28^ The voltammogram of Au-carbene-Fc surface (Figure 2B), obtained by decreasing the scan rate *v* from 5 to 0.05 V/s, displays well-defined asymmetric and reversible redox peaks. Both the anodic peak current *I*_pa_ and the cathodic peak current *I*_pc_ decrease as the scan rate decreases. Furthermore, Figure 2C shows the plot of *I*_pa_ and *I*_pc_ as a function of *v*, along with linear fittings with a correlation coefficient of *R*^2^ > 0.99. This linear *I*–*v* behavior indicates that the Fc moieties are surface immobilized, confirming the covalent coupling of Fc to the carbene surface.

When the concentration of the *C*_diazo_ is increased from 0.1 to 0.2 mg/mL, the thickness of the carbene thin film on the Au surface changes from 1.60 ± 0.10 to 2.34 ± 0.14 nm, as measured by ellipsometry. This relationship between precursor concentration and carbene thin film thickness is attributed to the polymerization and cross-linking of carbene with −NH_2_ groups during surface modification.^67^ After Fc attachment, for both concentrations, the shapes of the redox peaks in the cyclic voltammogram only show a slight increase in peak height (Figure 2D), indicating that the amount of attached Fc does not increase as the concentration of carbene increases. Additionally, the shoulder peak at 0.59 V becomes more pronounced at higher *C*_diazo_. This phenomenon is likely due, in part, to the increased surface polymerization and cross-linking of carbene with −NH_2_ groups as *C*_diazo_ increases,^67^ resulting in formation of more N-substituted amino group but with a lower-than-expected increase in the number of exposed −NH_2_ groups (see Figure S9 for the −NH– groups). Peak fitting (Figure S6) was conducted for the calculation of the surface coverages Γ_Fc_ (mol/cm^2^) (Table 1, estimated from eq S1). The Γ_Fc_ value for all carbene concentrations is similar, approximately 0.25 times the theoretical value of 4.5 × 10^–10^ mol/cm^2^ for ferrocenylalkanethiolated SAMs.^68^ By dividing the Γ_Fc_ by the carbene grafting density estimated from XPS measurements, a coupling efficiency of 54% was determined (see Supporting Information Section S3 for details on the carbene density calculation). The lower Fc coverage is therefore attributed to incomplete coupling, bulky structure of the bis(diaryldiazomethane) system, and the deposition conditions for the carbene thin film,^51^ in contrast to the compact size and solution growth of densely packed thiolated SAMs.

**Table 1:** Measured Properties of Au-Carbene-Fc Surface at Different Precursor Concentration of *C*_diazo_

*C*_diazo_ (mg/mL)	*E*_pa_ (mV)	*E*_pc_ (mV)	Δ*E*_p_ (mV)	*E*_HOMO_ (eV)	Γ_Fc_ (× 10^–10^ mol/cm^2^)
0.1	328 ± 3	260 ± 1	69 ± 2	–4.99 ± 0.02	1.08 ± 0.01
0.2	349 ± 3	265 ± 4	84 ± 1	–5.00 ± 0.02	1.37 ± 0.15

### 2.3 Charge Transport Measurements of the Molecular Rectifier

**Figure 3:**
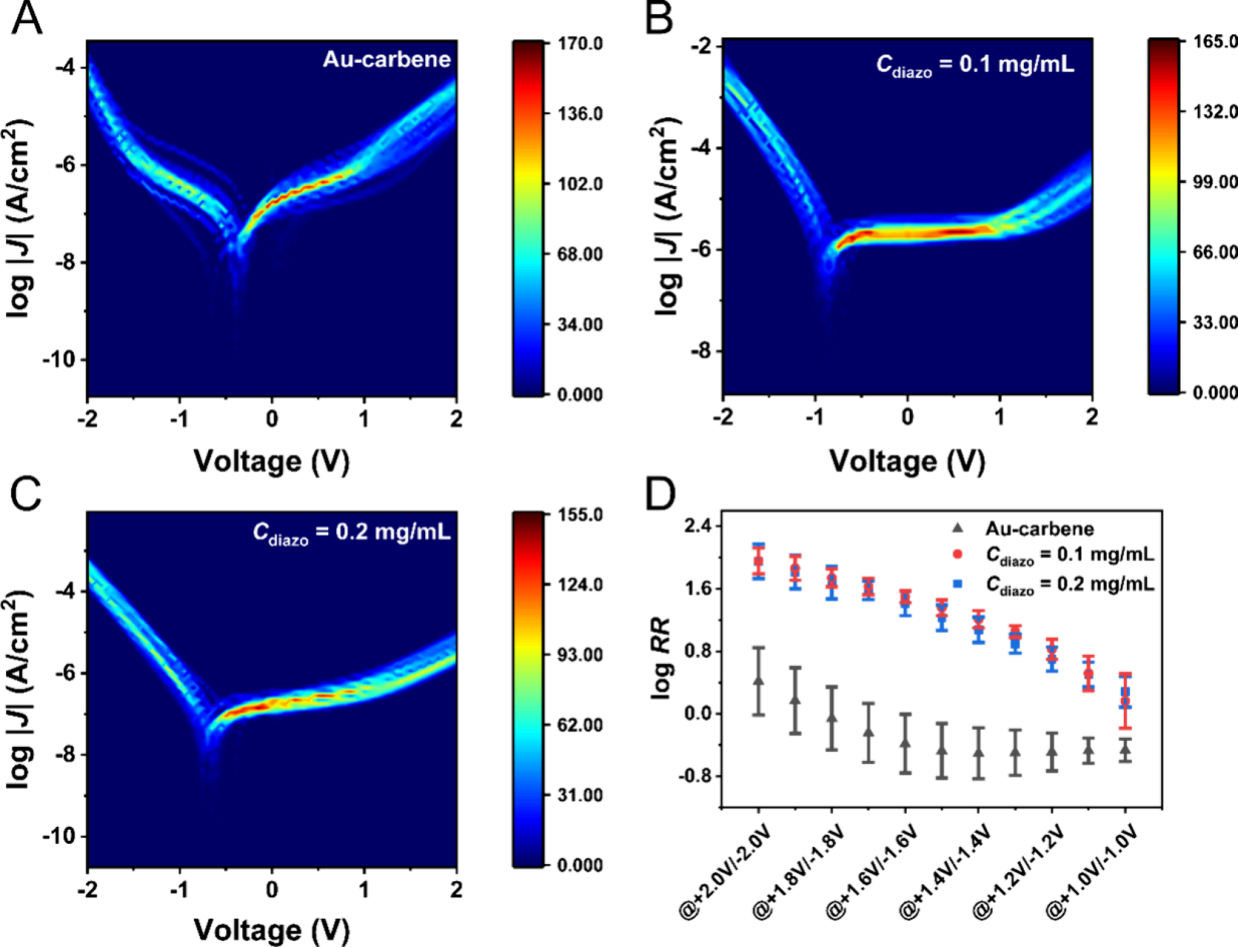
Electrical characteristics of the EGaIn junction. (A) Heatmap illustration of the Log *J*(*V*) behavior for the Au-carbene//EGaIn junction where *C*_diazo_ is 1.0 mg/mL, this junction serves as a control, and the high *C*_diazo_ is used due the high voltage applied. (B) Heatmap illustration of the Log *J*(*V*) behavior for the Au-carbene-Fc//EGaIn junction where *C*_diazo_ is 0.1 mg/mL. (C) Heatmap illustration of the Log *J*(*V*) behavior for the Au-carbene-Fc//EGaIn junction where *C*_diazo_ is 0.2 mg/mL. Note that the offset of *J*(*V*) curves with respect to *V* = 0 has been explained in detail due to either capacitive charging, an electrochemical process, or conformational rearrangement of the molecular layer.^7,77^ (D) Plot of Log RR determined at ±2.0 to ±1.0 V at an interval of 0.1 V as a function of *C*_diazo_. The error bars for all represent standard deviations from ∼30 junctions.

Figure 3B,C shows log |*J*| (current density) versus *V* curves of the Au-carbene-Fc//EGaIn junction at two carbene concentrations, where the two cases exhibit current rectification behavior with RR values of 90.6 and 91.2 at ±2.0 V. As a reference sample, the Au-carbene//EGaIn junction without Fc modification was also measured (Figure 3A), which does not rectify (RR = 2.6 at ±2.0 V). This clearly demonstrates that the rectification behavior of the Au-carbene-Fc//EGaIn junction is caused by the attachment of Fc rather than the carbene on the electrodes. The rectification performance of Fc-based junctions can be affected by many factors, such as molecular conformation,^69^ bottom electrode roughness,^70^ and thiol stability^39^ (see more in Supporting Information Section S8). Gauche defects exist in the carbene layer, and the Fc moiety on Au-carbene-Fc is likely disordered (Figure S9), which leads to lower rectification as compared with the current rectification on gold substrates for tunneling junctions containing Fc listed in Table S2.

The current density at positive voltage (i.e., reverse bias direction) decreases with increasing *C*_diazo_ as shown in Figure 3B,C. This trend can be well explained by an increased tunneling barrier at the higher concentration. Interestingly, the rectification ratios are similar at the two carbene concentrations (Figure 3D). Since the activation voltage of the molecular rectifier is typically correlated with the energy difference between the conduction molecular orbital (specifically, the HOMO) and the electrode, this similarity in RR indicates consistent HOMO energy alignment for both junctions. Table 1 shows that the *E*_HOMO_ (the energy of the highest occupied molecular orbital), estimated from CV using eq S3, is nearly identical for the two carbene concentrations, which aligns with the observed rectification behavior.

### 2.4 Stability of the Molecular Rectifier

**Figure 4:**
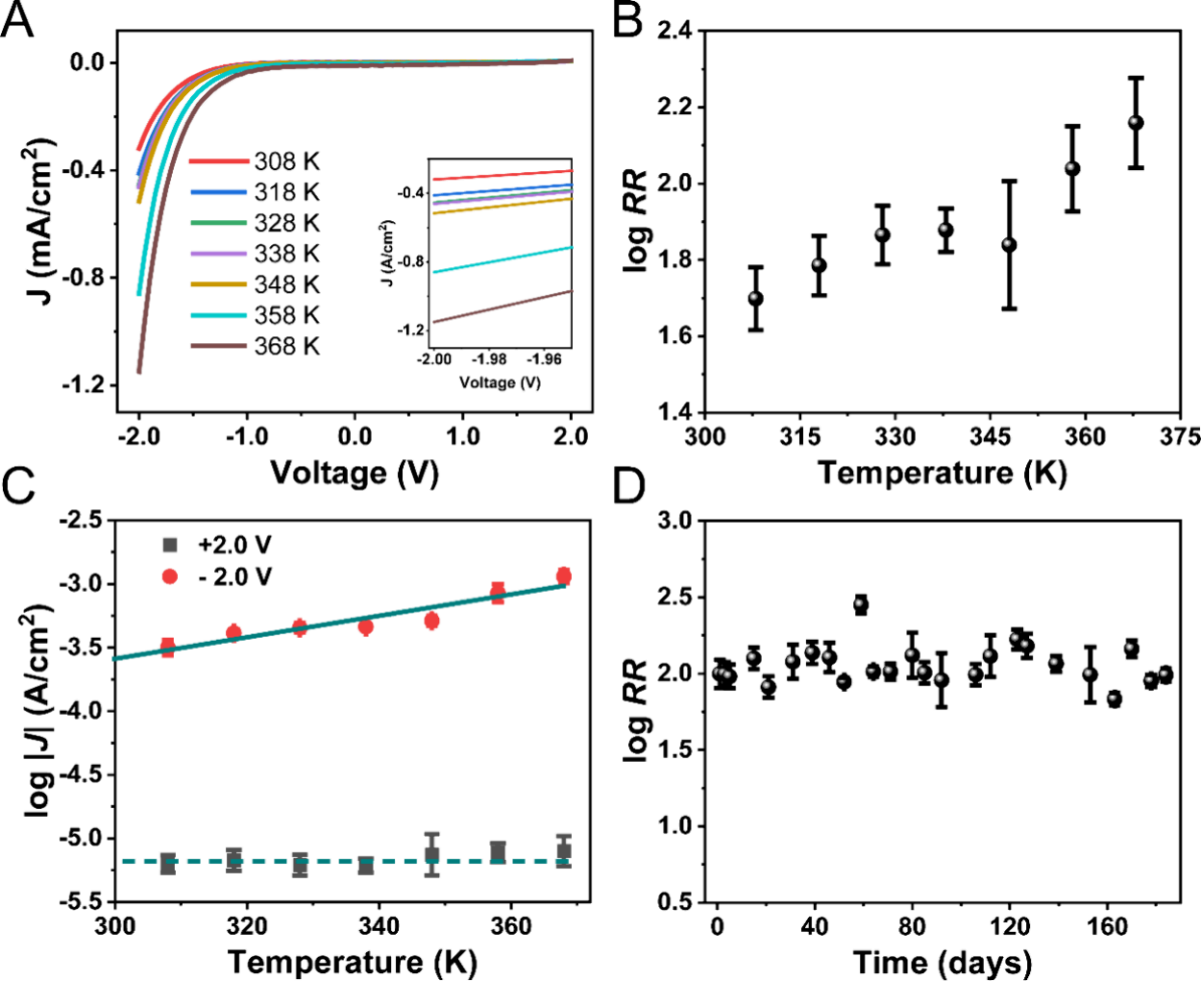
Stability characteristics of the Au-carbene-Fc//EGaIn junction. (A) *J*(*V*) curves for the Au-carbene-Fc//EGaIn junction recorded over a temperature range of 308–368 at 10 K increments for *C*_diazo_ = 0.1 mg/mL. Inset: magnified view around a bias of −2.0 V. (B) Log RR determined at ±2.0 V as a function of temperature ranging from 308 to 368 K. C) Plot of log |*J*| at ±2.0 V as a function of temperature (308–368 K). The solid cyan line represents a linear fit to eq 1 for data collected at −2.0 V, while the dashed cyan line indicates a horizontal guide for the data collected at +2.0 V. (D) Stability of the Log RR at ±2.0 V for the Au-carbene-Fc//EGaIn junctions measured over a period of 180 days. In panel (B), error bars indicate standard deviations from 20 scans at the same location, while in panel (D), error bars represent standard deviations from multiple measurement positions across the same sample.

Given the higher bonding energy of the Au–C bond relative to the Au–S bond, carbene-based molecular rectifiers are expected to exhibit enhanced thermal and storage stability. Figure 4A shows the temperature-dependent measurement of the Au-carbene-Fc//EGaIn junction. Different from the previous temperature-dependent measurement on SC_*n*_Fc SAMs, which reached 330 K,^71^ the carbene-based junction enabled a higher temperature range of 523–605 K.^49,50,72^ As the temperature increases from 308 to 368 K, the positive currents are independent of temperature corresponding to coherent tunneling, while the negative currents increase clearly with the temperature, indicating that the charge transport is thermally activated (see Figure S7 for the charge transport mechanism). As a result, the RR value also increases from ∼41 to ∼144 with increasing temperature (Figure 4B). The activation energy for hopping (*E*_a_) was determined by fitting to eq 1, where *k*_b_ is the Boltzmann constant, *T* is the temperature, *J*_0_ is the pre-exponential factor, and *J* is the current density at the measured temperature.
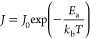
1

From the slope of the Arrhenius plot (Figure 4C), the value of *E*_a_ is 63 ± 14 meV, which is comparable to that of SC_*n*_Fc junctions,^73^ confirming hopping is the dominant charge transport mechanism at negative bias. Further, we also followed the rectification behavior of the Au-carbene-Fc//EGaIn junction over a period of 6 months. The Au-carbene-Fc substrate was stored under ambient conditions. As depicted in Figure 4D (and Supporting Information Section S9), the RR values at ±2.0 V remain high, with an average value of 122.6 ± 42.5 over 6 months. In contrast to the short lifespan in thiolated SAM-based junctions, the Au-carbene-Fc-based rectifiers demonstrate enhanced durability, which is an important advantage of these molecular rectifiers.

## 3 Conclusions

In summary, we demonstrated a convenient method of preparing molecular rectifiers with rectification ratios of ∼100 by one-step covalent bonding of a ferrocene unit onto carbene-modified gold substrates. The thin films on gold were characterized by XPS and electrochemical measurements, confirming the successful chemical attachment of ferrocene. Our findings suggest that the surface coverage of Fc does not vary significantly, while the rectification ratio remains unchanged when the carbene thin film becomes thicker. Moreover, temperature-dependent measurement up to 368 K shows a hopping mechanism of charge transport at negative bias, with an activation energy comparable to that of SC_*n*_Fc junctions. Notably, the electrical properties and rectification ratio of the ferrocene attached carbene thin film are stable for 6 months of storage under ambient conditions. Therefore, the simple method to attach functional groups onto a highly stable carbene surface provides a new pathway for the design and promising application of durable functional molecular electronic devices.

## 4 Experimental Section

### 4.1 General Information

All of the chemicals and solvents were used directly as supplied by Sigma-Aldrich or Sinopharm Chemical Reagent Co. Ltd. The carbene precursor—bis(diaryldiazomethane) terminated with −NH_2_ (Figure 1A) was synthesized according to the previous report (see also Supporting Information Section S1).^67^ A 100 nm layer of gold (Au) was deposited onto the silicon (Si) substrate, with a 5 nm chromium (Cr) layer serving as the adhesion layer. Two different concentrations of bis(diaryldiazomethane) in dichloromethane (DCM) (denoted as *C*_diazo_ = 0.1 and 0.2 mg/mL) were chosen. Following the immersion of the Au substrate into the solution for 5 s, the sample was subsequently heated at 120 °C for 30 min, and the Au-carbene surface was washed extensively with ethanol and DCM and dried under N_2_ for further use.

### 4.2 Covalent Coupling of FcCH_2_COOH onto the Carbene-Based Thin Film

The covalent attachment of Fc derivative was accomplished through carbodiimide coupling.^74,75^ Initially, a solution of 5 mM FcCH_2_COOH in water was sonicated and filtered to obtain a clear solution. Subsequently, 5 mM EDC (1-ethyl-3-(3-dimethylamino) propyl carbodiimide) in water was added at 4 °C. The Au-carbene surface was then immersed in the solution for 48 h, followed by thorough washing with DI water, ethanol, and DCM, and finally dried with N_2_ to obtain Au-carbene-Fc surface. N_2_ protection was employed throughout the entire process to prevent potential oxidation.

### 4.3 Characterization of the Modified Au Surface

(1) The X-ray photoelectron spectroscopy (XPS) spectra were acquired by utilizing the ESCALAB 250Xi from ThermoScientific. A monochromatic Al Kα source with a photo energy of 1486.6 eV was employed under a vacuum condition (10^–9^ mbar). The pass energy was 100 and 30 eV for the full and high-resolution spectra, respectively. A normal takeoff angle at 90° and the X-ray spot size of 500 μm were used. The spectra were analyzed by Avantage 5.9925 (Thermo Fisher Scientific) and calibrated by Au 4*f*_7/2_ peak at 84.0 eV. (2) The modified Au electrodes were characterized by cyclic voltammetry using a BioLogic SP-200 Potentiostat. A custom-built electrochemical cell was equipped with Pt as a counter electrode, Ag/AgCl as a reference electrode was used, and modified Au work electrode was exposed to aqueous 1.0 M HClO_4_ at an area of 0.28 cm^2^. Voltage was applied between 0.1 and 0.9 V at a scan rate of 0.05 to 5 V/s. The peak fitting and plotting were done by Origin 2022b software. (3) The thickness of Au-carbene at 0.1 and 0.2 mg/mL was measured by an optical ellipsometer (M-2000v from J.A. Woollam) at an incidence angle of 70°. (4) Atomic force microscopy (AFM) images were recorded with Bruker Dimension Icon using tapping mode at a measured area of 1 × 1 μm^2^, and the surface roughness of the Au surface was determined.

### 4.4 Electrical Measurements

(1) The EGaIn (eutectic metal alloy with 75.5% Ga and 24.5% In) technique was used for all junction measurements, with freshly prepared Ga_2_O_3_/EGaIn cone tip as the top electrode and modified Au surface as the bottom electrode. Voltage was applied in the range of ±2.0 V, 20 traces of *J*(*V*) curves was recorded for each junction, and statistical analysis was based on over 30 junctions. The average log |*J*|, log RR, and log standard deviation values of each measurement were obtained with Gaussian fitting to the histograms from statistical analysis. (2) For the temperature-dependent measurement, the Au substrate was specifically fabricated.^76^ SiO_2_ microspheres with a diameter of 30 μm (Suzhou Knowledge & Benefit Sphere Tech. Co., Ltd.) were first dispersed onto the Au substrate. Al_2_O_3_ (50 nm) was then deposited on the substrate by electron beam evaporation to form an insulating layer. Finally, the microspheres were removed from the substrate with a stream of N_2_ gas. A micropore was created for the confinement of the EGaIn top electrode. After the EGaIn top electrode was brought into contact with the Au-carbene-Fc surface, the junction was measured while the surface was heated in situ from 308 to 368 K in 10 K intervals. (3) For stability measurement, the electrical properties of an Au-carbene-Fc substrate were monitored for over 180 days while stored in ambient conditions. Each measurement was based on the analysis of 2–3 junctions.

## Supporting Information Available

Additional synthesis of the carbene precursor, AFM characterization, XPS characterization and carbene grafting density calculation, electrochemical characterization, energy level diagrams for Au-carbene-Fc//EGaIn junction, electrical characteristics of the junctions, and factors affecting the rectification of Fc in tunneling junctions and stability of the Fc-functionalized carbene-based molecular diodes (PDF)
