# Supplementary material for: A Robust Molecular Rectifier Based on Ferrocene-Functionalized Bis(diarylcarbene) on Gold
Source: ACS Appl Mater Interfaces. 2025 Feb 17;17(8):12875–82. doi: 10.1021/acsami.4c20999 (PMC11873912; doi:10.1021/acsami.4c20999)
Supplement: Supplementary file 1 — am4c20999_si_001.pdf [file am4c20999_si_001.pdf]

Supporting information

A Robust Molecular Rectifier Based on Ferrocene Functionalized Bis(diarylcarbene) on Gold

Dandan Wang [a§], Wenrui Xu [b§], Yidan Hu[b], Tao Wang [b*], Mark G. Moloney [a,c*], Wei Du [b*]

[a]Oxford Suzhou Centre for Advanced Research, Building A, 388 Ruo Shui Road, Suzhou Industrial Park, Jiangsu, 215123, P.R. China

[b]Institute of Functional Nano & Soft Materials (FUNSOM), Jiangsu Key Laboratory for Carbon-Based Functional Materials & Devices, Soochow University, 199 Ren'ai Road, Suzhou, 215123, Jiangsu, P. R. China

[c]Chemistry Research Laboratory, Department of Chemistry, University of Oxford, Oxford OX1 3TA, U.K.

[*]E-mail: duwei2021@suda.edu.cn; mark.moloney@chem.ox.ac.uk; wangtao2019@suda.edu.cn

[§]*D.W. and W.R. contributed equally to this work*

S1. Synthesis of the carbene precursor

Figure S1 shows the synthetic route for the carbene precursor--bis(diaryldiazomethane) terminated with -NH_2. The detailed procedure have been published in literature,[1,2] 4-phenoxyaniline was used as the starting reagent, followed by four steps synthesis, the carbene precursor 4,4'-(((1,3-phenylenebis(diazomethylene)) bis(4,1-phenylene)) bis(oxy))dianiline was obtained as a dark red solid. The proton NMR spectra recorded by JEOL 400 M spectrometer for the compound in each step were shown in **Figure S2**.

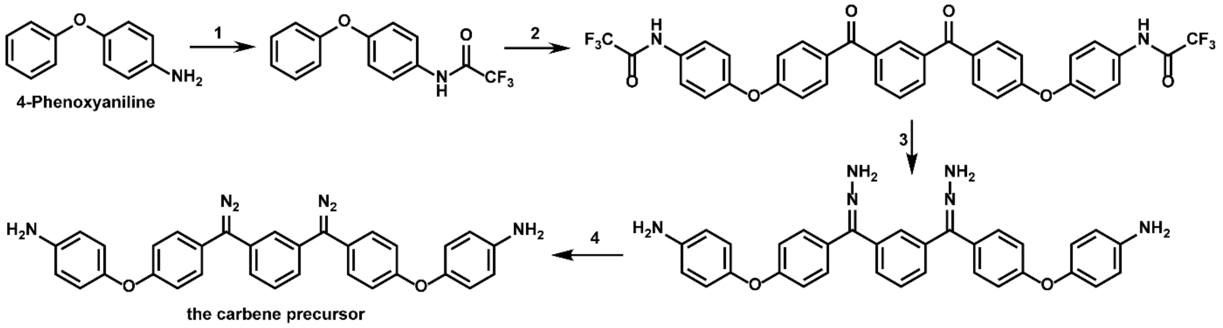

Figure S1. The synthetic route for the carbene precursor.

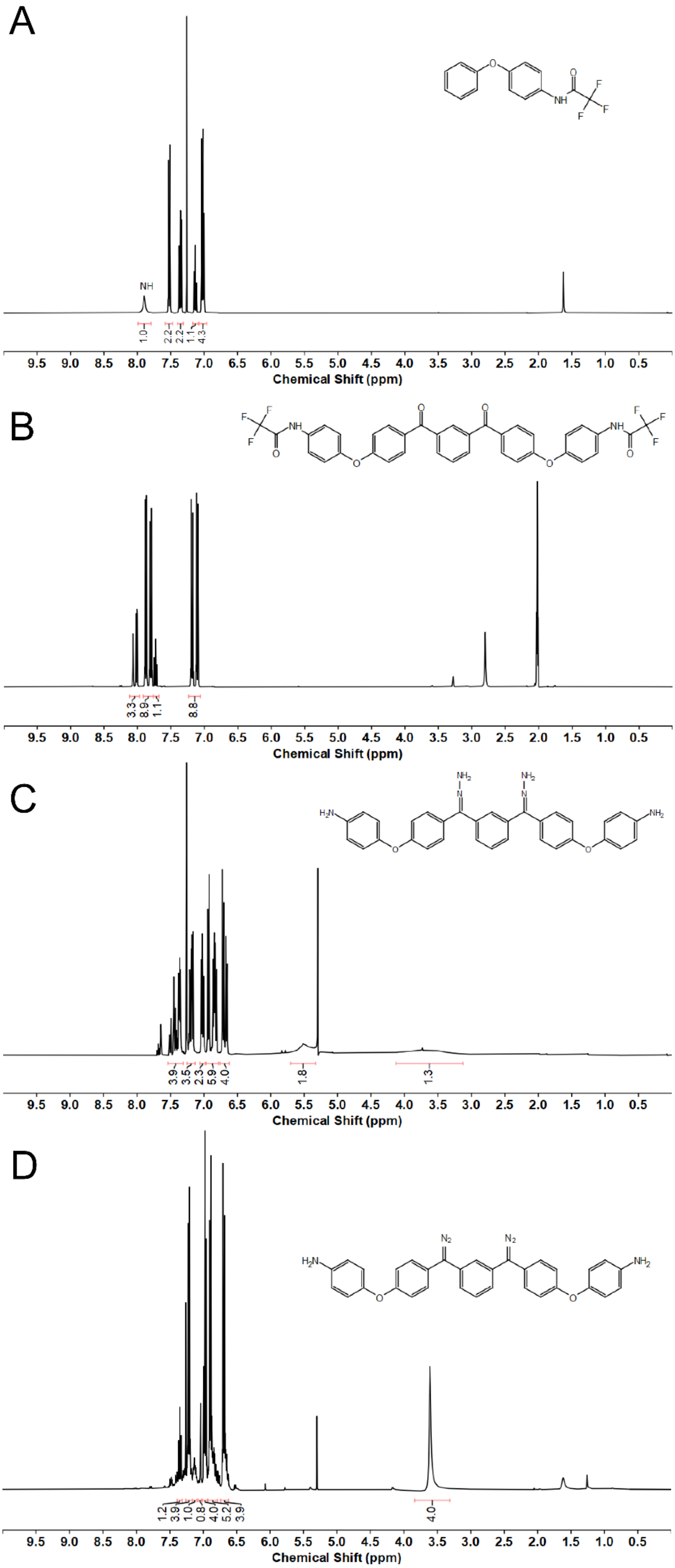
A
NH
1.0
2.2
2.2
1.1
4.3
9.5 9.0 8.5 8.0 7.5 7.0 6.5 6.0 5.5 5.0 4.5 4.0 3.5 3.0 2.5 2.0 1.5 1.0 0.5
Chemical Shift (ppm)
B
3.3
8.9
1.1
8.8
9.5 9.0 8.5 8.0 7.5 7.0 6.5 6.0 5.5 5.0 4.5 4.0 3.5 3.0 2.5 2.0 1.5 1.0 0.5
Chemical Shift (ppm)
C
3.9
3.5
2.3
5.9
4.0
1.8
1.3
9.5 9.0 8.5 8.0 7.5 7.0 6.5 6.0 5.5 5.0 4.5 4.0 3.5 3.0 2.5 2.0 1.5 1.0 0.5
Chemical Shift (ppm)
D
1.2
3.9
1.0
0.8
4.0
5.2
3.9
4.0
9.5 9.0 8.5 8.0 7.5 7.0 6.5 6.0 5.5 5.0 4.5 4.0 3.5 3.0 2.5 2.0 1.5 1.0 0.5
Chemical Shift (ppm)

Figure S2. ^{1}H NMR spectra for the compound generated each step. A) 2,2,2-trifluoro-N-(4-phenoxyphenyl) acetamide in $CDCl_3$. B) N,N'-(((isophthaloyl bis(4,1-phenylene))bis(oxy)) bis(4,1-phenylene)) bis(2,2,2-trifluoroacetamide) in $(CD_3)_2CO$. C) 4,4'-(((1,3-phenylene bis(hydrazineylidenemethylene)) bis(4,1-phenylene)) bis(oxy)) dianiline in $CDCl_3$. D) 4,4'-(((1,3-phenylene bis(diazomethylene)) bis(4,1-phenylene)) bis(oxy)) dianiline in $CDCl_3$.

S2. AFM Characterization

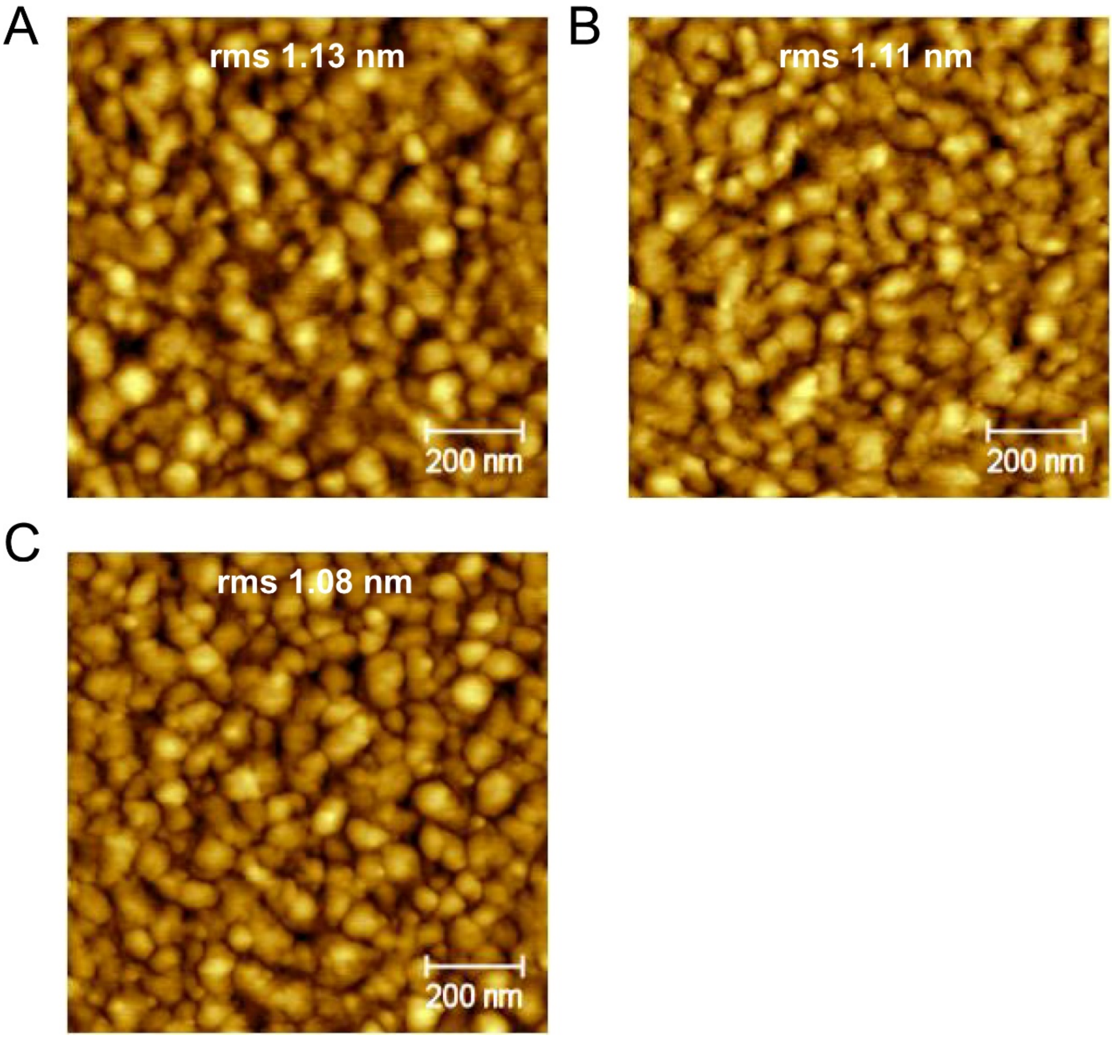

Figure S3. AFM image of A) the Au surface. B) Au-carbene with C_{diazo} = 0.10 mg/mL and C) Au-carbene with C_{diazo} = 0.20 mg/mL.

S3. XPS characterizations and carbene grafting density calculation.

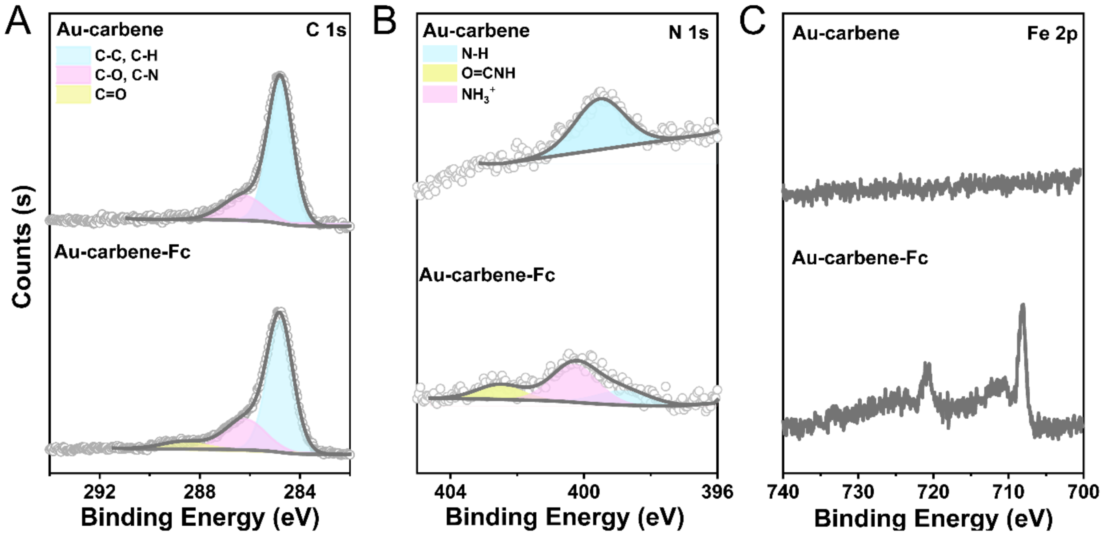

Figure S4. XPS characterizations of the Au-carbene and Au-carbene-Fc surface prepared with C_{diazo} = 0.20 mg/mL. High resolution spectroscopy of A) C 1s. B) N 1s. C) Fe 2p.

Figure S4 shows the high resolution XPS comparison of Au-carbene and Au-carbene-Fc surface at C_{diazo} = 0.20 mg/mL. The data confirm the successful coupling of Fc onto the Au-carbene surface and reveal the presence of unreacted amino moieties as indicated by the N 1s peak. These findings are consistent with the results obtained from the CV measurements.

To assess potential contamination of the carbene surface by the coupling reagent EDC, an Au-carbene substrate was immersed in an aqueous EDC-only solution under the same conditions used for Fc coupling (substrate: Au-carbene + EDC). The overlapping high-resolution XPS spectra of C 1s, N 1s, and O 1s for the two substrates, shown in **Figure S5**, exhibit close similarity, indicating no significant EDC contamination on the carbene surface.

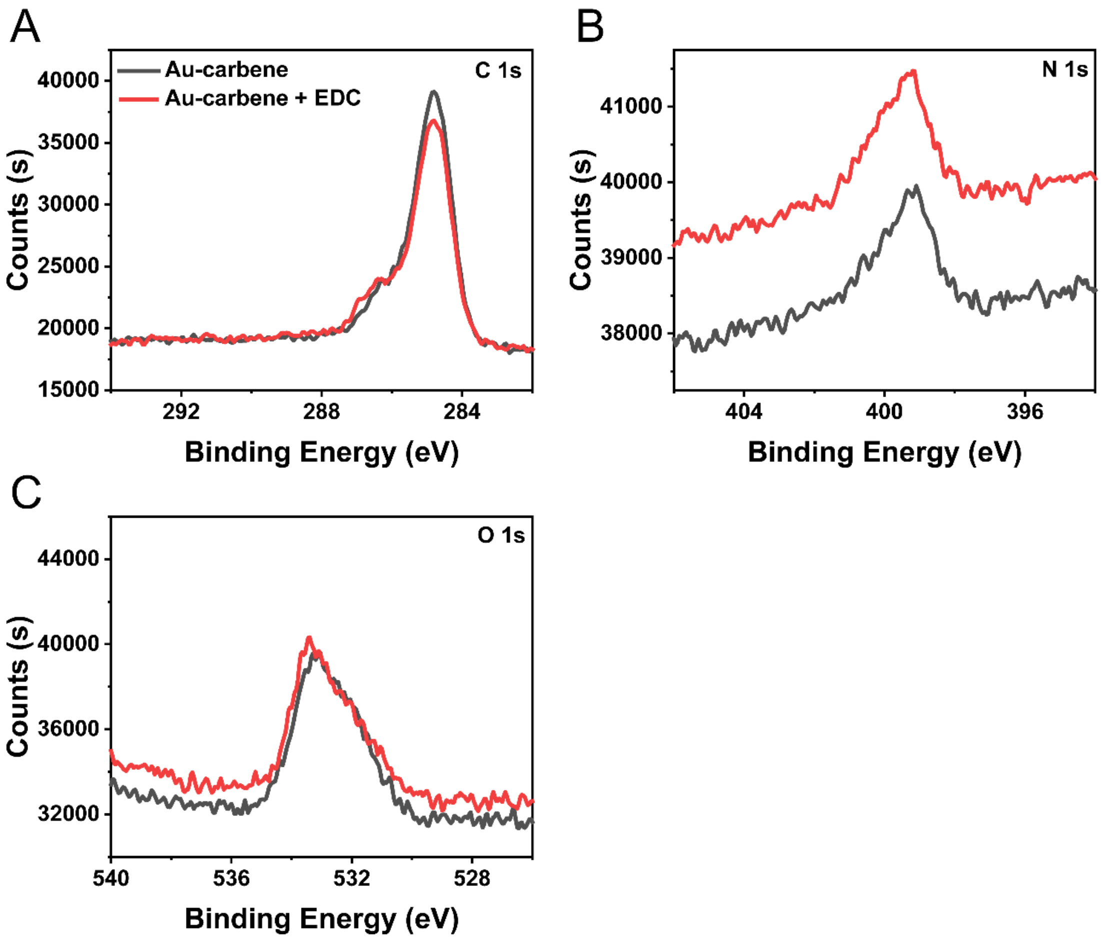

Figure S5. Comparison of XPS high resolution spectroscopy for A) C 1s. B) N 1s and C) O 1s. The black curve is the control Au-carbene substrate, and the red curve is the Au-carbene + EDC substrate.

To evaluate the efficiency of the Fc coupling reaction, the carbene grafting density was estimated by assuming that all N atoms contribute equally to the intensity of N 1s peak. The number density of the carbene layer (n_N) can be determined from the ratio of the spectral intensity of N 1s from the carbene layer (I_N) and Au 4f from the bare gold (I_{Au}), as shown in **Equation S1**:[3,4]

$$\frac{I_N}{I_{Au}} = \frac{n_N S_N}{P_N \int_0^{\infty} D_{Au} S_{Au} e^{-x/\lambda_{Au}} dx} = \frac{n_N S_N}{P_N D_{Au} S_{Au} \lambda_{Au}} \quad \text{(S1)}$$

where D_{Au} is the atomic density of Au in the bulk, λ_{Au} is the inelastic mean free path (IMFP) of Au photoelectrons through carbene layer [5] and S_N and S_{Au} are the atomic

surface sensitivity factors obtained by dividing the conventional bulk factors by the IMFP value.[6] P_N is the transmission probability through the carbene layer, and x is the distance below the surface.[7] n_N is calculated with the following set of variables: λ_{Au} = 4.2 nm at 1402.6 eV, λ_N = 2.8 nm at 1087.6 eV, S_N = 0.16, D_{Au} = 5.892 × 10^{22} atoms/cm^3,[4,5,7-9] and assuming each bound carbene molecule contains 2 N atoms, as shown in the structure in **Figure 1**A. **Table S1** shows the atomic percentage of c arbene modified surface and the final grafting density of carbene layers. Our value is consistent with previously reported carbene modification densities of 10^{13} to 10^{14} atoms/cm^2.[3,4,10,11] Considering the surface coverage of Fc in **Table 1**, the coupling efficiency of Fc onto Au-carbene is estimated to be ~54%. This incomplete coupling is likely due to the steric hindrance from bulky Fc molecules, cross-linking of carbene molecules reducing available free $-NH_2$ sites, and potential inaccuracies in structural assumptions from the XPS calculation.

Table S1. Atomic percentage for carbene modified surface and the grafting density estimated from XPS

Substrate	C_{diazo} (mg/mL)	Atomic percentage (%) C	N	O	Au	n_N (atoms/cm^2)	Grafting density (mol/cm^2)
Au-carbene	0.1	37.7	2.2	15.5	44.7	2.5 × 10^{14}	2.0 × 10^{-10}
	0.2	47.0	3.1	16.2	33.7	3.0 × 10^{14}	2.5 × 10^{-10}

S4. Electrochemical Characterization

We calculated the surface coverage Γ_{Fc} (mol/cm^2) according to **Equation S2**, where Q_{tot} is the total charge integrated from the anodic wave of CV, n is the number of electrons per mole of reaction (here n = 1), F is the faraday constant (96485 C/mol), and A is the surface area of the working electrode exposed to the electrolyte (here A =

0.28 cm^2).

$$\Gamma_{Fc} = \frac{Q_{tot}}{nFA} \tag{S2}$$

$$E_{HOMO} = E_{abs,NHE} - eE_{1/2,NHE} \tag{S3}$$

We determined the energy level of the highest occupied molecular orbital (E_{HOMO}) by **Equation S3**, where $E_{\text{abs,NHE}}$ is the absolute potential energy of the normal hydrogen electrode (NHE, -4.5 eV), $E_{1/2,\text{NHE}}$ is the formal half-wave potential versus the NHE by average E_{pa} and E_{pc} from CV. The values have been summarized in **Table 1**.

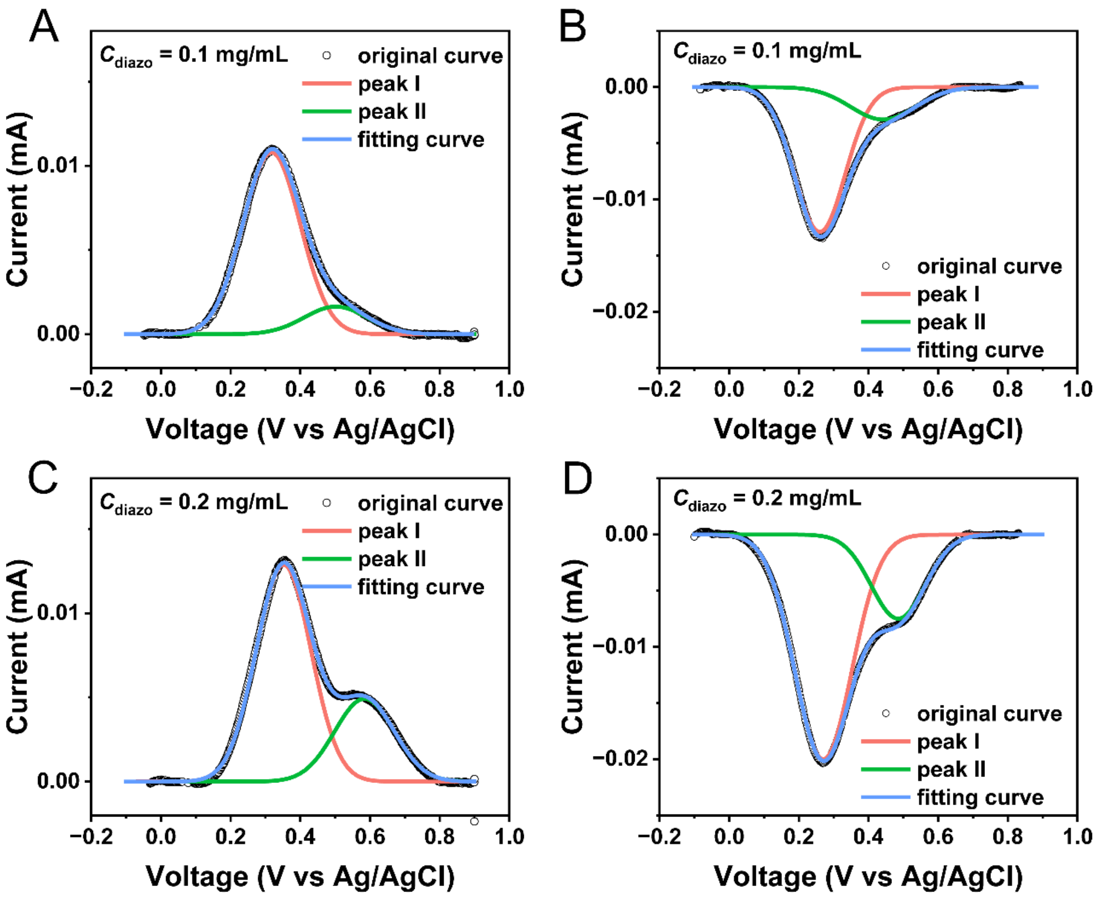

Figure S6. A-B) Anodic and cathodic peak fitting for C_{diazo} = 0.1 mg/mL. C-D) Anodic and cathodic peak fitting for C_{diazo} = 0.2 mg/mL.

The anodic and cathodic peaks for C_{diazo} = 0.1 and 0.2 mg/mL were fitted with two gaussian peaks using Origin. **Figure S6** shows the fitted peaks. All the surface coverage was calculated using the area of peak I.

S5. Energy level diagram for Au-carbene-Fc//EGaIn junction

For the Au-carbene-Fc/EGaIn junction, the highest occupied molecular orbital (HOMO) is localized on the Fc moieties.[12,13] **Figure S7** shows that under a bias of -2.0 V, the HOMO falls within the conduction window, enabling charge hopping via the Fc groups. While at +2.0 V, the HOMO lies outside the conduction range and does not participate in charge transport.

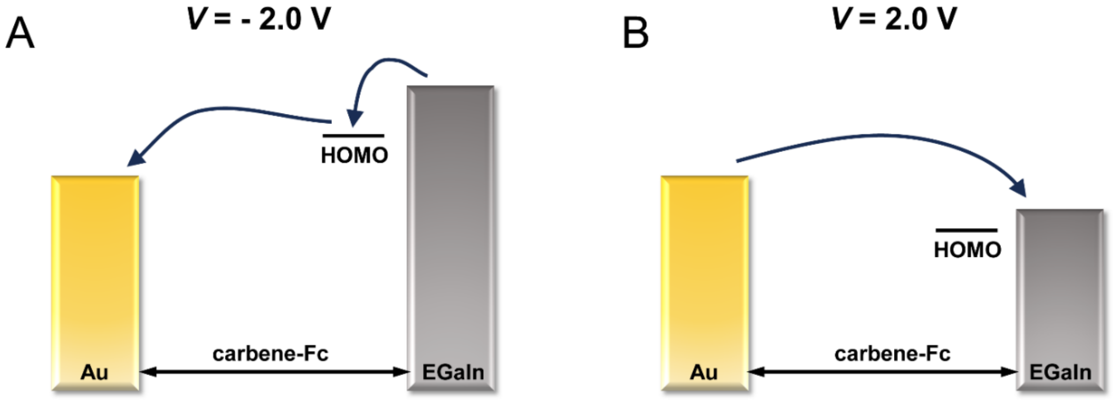

Figure S7. Energy level diagrams of the Au-carbene-Fc//EGaIn junction at a bias of A) -2.0 V and B) +2.0 V. The curved arrows indicate the electron transport pathway.

S6. Electrical characteristics of the junctions

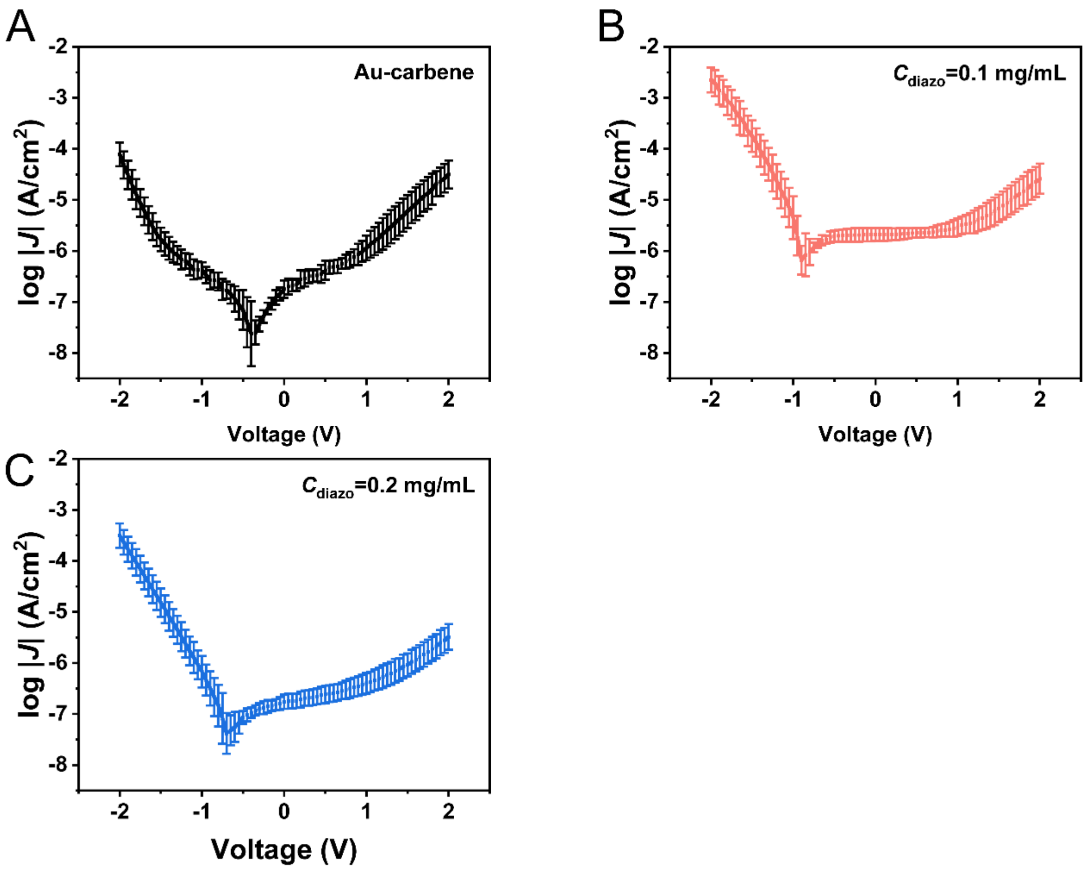

Figure S8. Electrical characteristics of the tunnelling junctions of A) Au-carbene, B) Au-carbene-Fc with C_{diazo} = 0.1 mg/mL, C) Au-carbene-Fc with C_{diazo} = 0.2 mg/mL. The error bars represent the log standard deviations obtained from the Gaussian fitting of the histograms.

S8. Factors affecting the rectification of Fc in tunnelling junctions

Molecular conformation has been reported to be critically matters for the rectification.[14] For our carbene system, we assume that the carbene modification on gold involves both carbene insertion and crosslinking on the gold surface.[15] **Figure S9** gives a schematic illustration of Fc on carbene surface, while some regions of the

carbene layer could potentially form a self-assembled monolayer (SAM), the overall carbene layer is less ordered compared to thiolated SAMs or N-heterocyclic carbene (NHC) SAMs. Consequently, the Fc moiety within our system is likely also disordered with existence of gauche defects.

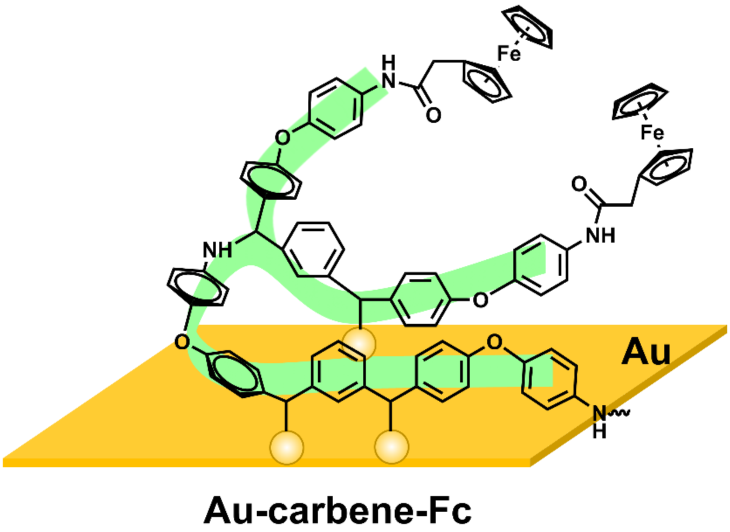

Figure S9. A schematic illustration of Fc coupling onto the carbene modified gold surface.

Furthermore, an increase in the roughness of the bottom metal substrate also reduces the Fc rectification.[16] To summarize, **Table S2** presents the rectification results for EGaIn junctions containing Fc on gold substrates. In our study, a rectification ratio (*RR*) of ~100 at ±2.0 V was observed, which is lower compared to reports such as: *RR* of 400-600 on Au^{TS} at ±1.0 V for Fc decorated triptycene-based tripods,[17] *RR* of ~1000 on thermally evaporated Au at ±1.0 V for Fc terminated metallocene based junctions,[18] *RR* of ~1000 on Au^{TS} at ±1.0 V for bi-Fc based junction,[19] and *RR* of ~2100 on Au^{TS} at ±1.0 V for Fc substituted thiofluorene junction.[20] However, our *RR* is higher than values reported for some systems, such as *RR* < 10 on Au^{TS} at ±1.5 V for 11-(ferrocenyl) undecanethiol based junctions,[21] and *RR* of 0.5-11 on Au^{TS} at ±1.0 V for SC_nFc based

junction.[22] The as-deposited gold substrate used in this study had an initial roughness of ~1.1 nm, which does not change significantly after low concentrations of carbene modification as measured by atomic force microscopy. This roughness, although higher than that of templated-stripped Au (Au^{TS}), remains low to prevent significant negative effects on junction quality during measurement.[18,23]

Table S2. A summary of rectification and gold substrate parameters for tunneling junctions containing Fc.

RR value	Fc structure	Au substrate		Ref
		preparation method	rms (nm)	
400-600 at ±1.0 V	Ferrocene-Decorated Triptycene-Based Tripod	template stripped (TS)	0.8	17
~1000 at ±1.0 V	Fc terminated metallocene	as deposited	0.8	18
~1000 at ±1.0 V	Fc-C≡C-Fc terminated	TS	0.4	19
~2100 at ±1.0 V	Fc substituted thiofluorene	TS	0.06	20
< 10 at ±1.5 V	$SC_{11}Fc$	TS	NA	21
0.5-11 at ±1.0 V	SC_nFc	TS	0.41	22
~100 at ±2.0 V	Fc terminated carbene layer	as deposited	1.1	this work

S9. Stability of the Fc-functionalized carbene-based molecular diode.

Thiol-based molecular junctions are known to exhibit instability due to oxidative degradation and desorption from the gold surface, which can result in variability in rectification performance.[24,25] In contrast, our carbene-based system provides enhanced stability due to the strong covalent bonding between the carbene moiety and the gold substrate.[26] To test the storage stability of the Fc-functionalized carbene surface, we followed the rectification behaviour of the same Au-carbene-Fc surface over a period

of six months. **Figure S10** shows the 3D plot of the Log $J(V)$ curves of the Au-carbene-Fc//EGaIn junctions as a function of time, where the *RR* values at ±2.0 V remains almost unchanged over six months, showing largely enhanced durability compared to SC_nFc junctions.

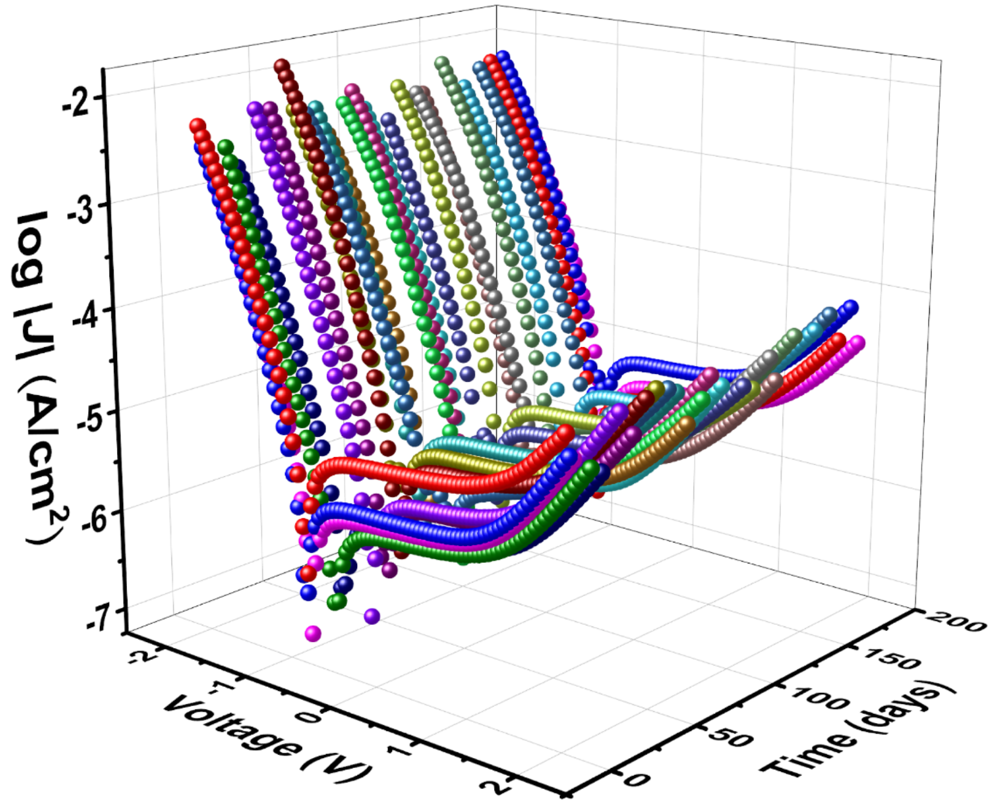

Figure S10. 3D plot of the Log J(V) curves for Au-carbene-Fc//EGaIn junctions prepared at 0.1 mg/mL.

On the other hand, we also followed the electrical characteristics and *RR* values of the same Au-carbene-Fc//EGaIn junction under the continuous operation over 1000 scans. As shown in **Figure S11**A, the *RR* value is stable over the first 100 traces, but slowly increases by ~120% during the subsequent 900 traces. Moreover, we also

visualized a decrease in current density at both -2.0 V and +2.0 V accompanied with the increasing *RR* value (**Figure S11**B). Such a time dependent decreasing of current density in EGaIn junctions have also been observed before,[27] which may be related to the mechanical stability of EGaIn junctions and due to the slowly loss of contact during the long-time electrical measurement. With the reduced contact, the defects in the molecular layer will have less effect on the charge transport, thus the reduction of the leakage current in the reverse bias direction will be more obvious than that in the forward bias direction. As a result, the *RR* value will slightly increase with the reduced contact, which explains our observation.

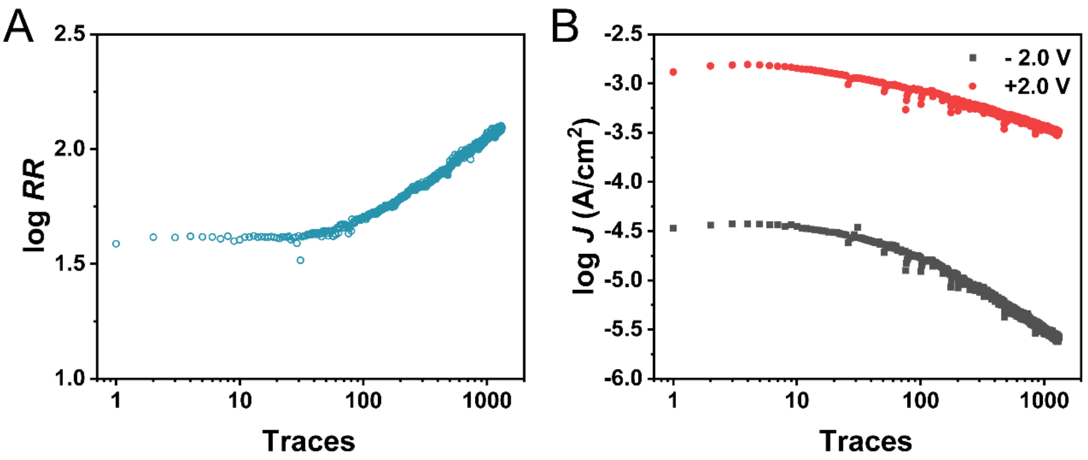

Figure S11. Electrical stability of the Au-carbene-Fc//EGaIn junction at 0.1 mg/mL with 1000 traces: A) Plot of *RR* as a function of the trace number. B) The current density *J* as a function of the trace number at -2.0 V and +2.0 V.

References

(1) Wang, D.; Hartz, W.; Christensen, K. E.; Moloney, M. G. Surface Modification of Glass Fiber Membrane via insertion of a bis(diarylcarbene) assisted with polymerization and cross-linking reactions. *Surf. Interfaces* **2022**, *32*, 102155.

(2) Moloney, M. G.; Yang, P. Surface Modification of Polymers with Bis(arylcarbene)s from Bis(aryldiazomethane)s: Preparation, Dyeing and Characterization. *RSC Adv.* **2016**, *6*, 111276.

(3) Wang, H.; Griffiths, J.-P.; Egdell, R. G.; Moloney, M. G.; Foord, J. S. Chemical Functionalization of Diamond Surfaces by Reaction with Diaryl Carbenes. *Langmuir* **2008**, *24*, 862-868.

(4) Neson, G. W.; Parker, E. M.; Singh, K.; Blanford, C. F.; Moloney, M. G.; Foord, J. S. Surface Characterization and *in situ* Protein Adsorption Studies on Carbene-Modified Polymers. *Langmuir* **2015**, *31*, 11086-11096.

(5) Bain, C. D.; Whitesides, G. M. Attenuation lengths of photoelectrons in hydrocarbon films. *J. Phys. Chem* **1989**, *93*, 1670-1673.

(6) Wagner, C. D.; Davis, L. E.; Zeller, M. V.; Taylor, J. A.; Raymond, R. H.; Gale, L. H. Empirical atomic sensitivity factors for quantitative analysis by electron spectroscopy for chemical analysis. *Surf. Interface Anal.* **1981**, *3*, 211-225.

(7) Briggs, D.; Seah, M. P. Practical Surface Analysis - Auger and X-ray Photoelectron Spectroscopy. Wiley, **1990**; pp 181-212.

(8) Petrovykh, D. Y.; Kimura-Suda, H.; Tarlov, M. J.; Whitman, L. J. Quantitative Characterization of DNA Films by X-ray Photoelectron Spectroscopy. *Langmuir* **2004**, *20*, 429-440.

(9) Petrovykh, D. Y.; Kimura-Suda, H.; Whitman, L. J.; Tarlov, M. J. Quantitative Analysis and Characterization of DNA Immobilized on Gold. *J. Am. Chem. Soc.* **2003**, *125*, 5219-5226.

(10) Iqbal, S.; Lui, Y.; Moloney, J. G.; Parker, E. M.; Suh, M.; Foord, J. S.; Moloney, M. G. A comparative study of diaryl carbene insertion reactions at polymer surfaces. *Appl. Surf. Sci.* **2019**, *465*, 754-762.

(11) Shepherd, C.; Hadzifejzovic, E.; Shkal, F.; Jurkschat, K.; Moghal, J.; Parker, E. M.; Sawangphruk, M.; Slocombe, D. R.; Foord, J. S.; Moloney, M. G. New Routes to Functionalize Carbon Black for Polypropylene Nanocomposites. *Langmuir* **2016**, *32*, 7917.

(12) Nerngchamnong, N.; Yuan, L.; Qi, D. C.; Li, J.; Thompson, D.; Nijhuis, C. A. The role of van der Waals forces in the performance of molecular diodes. *Nat. Nanotechnol.* **2013**, *8*, 113-118.

(13) Nijhuis, C. A.; Reus, W. F.; Barber, J. R.; Dickey, M. D.; Whitesides, G. M. Charge Transport and Rectification in Arrays of SAM-Based Tunneling Junctions. *Nano Lett.* **2010**, *10*, 3611-3619.

(14) Belding, L.; Root, S. E.; Li, Y.; Park, J.; Baghbanzadeh, M.; Rojas, E.; Pieters, P. F.; Yoon, H. J.; Whitesides, G. M. Conformation, and Charge Tunneling through Molecules in SAMs. *J. Am. Chem. Soc.* **2021**, *143*, 3481-3493.

(15) Xu, W. R.; Wang, D. D.; Guo, Q. Q.; Zhu, S.; Zhang, L.; Wang, T.; Moloney, M.

G.; Du, W. Robust Sub-5 Nanometer bis(Diarylcarbene)-Based Thin Film for Molecular Electronics and Plasmonics. *Adv. Mater.* **2023**, *35*, 2303057.
(16) Yuan, L.; Jiang, L.; Thompson, D.; Nijhuis, C. A. On the Remarkable Role of Surface Topography of the Bottom Electrodes in Blocking Leakage Currents in Molecular Diodes. *J. Am. Chem. Soc.* **2014**, *136*, 6554-6557.
(17) Liu, Y. B.; Sanjayan, S.; Shoji, Y.; Fukushima, T.; Zharnikov, M. Appearance of Different Conductance States in Monomolecular Films of Ferrocene-Decorated Triptycene-Based Tripods. *J. Phys. Chem. C* **2023**, *127*, 24458-24466.
(18) Asyuda, A.; Das, S.; Lang, H.; Zojer, E.; Zharnikov, M. Bias-Triggered Conductivity Switching and High Effective Rectification in Metallocene-Based Molecular Junctions. *Adv. Electron. Mater.* **2022**, *8*, 2200296.
(19) Chen, X. P.; Roemer, M.; Yuan, L.; Du, W.; Thompson, D.; del Barco, E.; Nijhuis, C. A. Molecular diodes with rectification ratios exceeding 10^5 driven by electrostatic interactions. *Nat. Nanotechnol.* **2017**, *12*, 797-803.
(20) Liu, Y. B.; Zojer, E.; Zharnikov, M. Sweep-Character-Dependent Switching of the Conductance State in Ferrocene-Substituted Thiofluorene Self-Assembled Monolayers. *ACS Appl. Mater. Interfaces* **2022**, *14*, 52499-52507.
(21) Nguyen, Q. V. Controlling Rectification in Metal-Molecules-Metal Junctions Based on 11-(Ferrocenyl) Undecanethiol: Effects of the Electronic Coupling Strength. *J. Phys. Chem. C* **2022**, *126*, 6405-6412.
(22) Yuan, L.; Thompson, D.; Cao, L.; Nerngchangnong, N.; Nijhuis, C. A. One Carbon Matters: The Origin and Reversal of Odd-Even Effects in Molecular Diodes with Self-Assembled Monolayers of Ferrocenyl-Alkanethiolates. *J. Phys. Chem. C* **2015**, *119*, 17910-17919.
(23) Ramin, L.; Jabbarzadeh, A. Effect of water on structural and frictional properties of self assembled monolayers. *Langmuir* **2013**, *29*, 13367-13378.
(24) Kong, G. D.; Yoon, H. J. Influence of Air-Oxidation on Rectification in Thiol-Based Molecular Monolayers. *J. Electrochem. Soc.* **2016**, *163*, G115-G121.
(25) Willey, T. M.; Vance, A. L.; van Buuren, T.; Bostedt, C.; Terminello, L. J.; Fadley, C. S. Rapid degradation of alkanethiol-based self-assembled monolayers on gold in ambient laboratory conditions. *Surf. Sci.* **2005**, *576*, 188-196.
(26) Crudden, C. M.; Horton, J. H.; Ebralidze, II; Zenkina, O. V.; McLean, A. B.; Drevniok, B.; She, Z.; Kraatz, H. B.; Mosey, N. J.; Seki, T.; et al. Ultra stable self-assembled monolayers of N-heterocyclic carbenes on gold. *Nat. Chem.* **2014**, *6*, 409-414.
(27) Yuan, L.; Breuer, R.; Jiang, L.; Schmittel, M.; Nijhuis, C. A. A Molecular Diode with a Statistically Robust Rectification Ratio of Three Orders of Magnitude. *Nano Lett.* **2015**, *15*, 5506-5512.
